# Racial and Ethnic Disparities in Hospital-Based Care Among Dual Eligibles Who Use Health Centers

**DOI:** 10.1089/heq.2022.0037

**Published:** 2023-01-13

**Authors:** Brad Wright, Jill Akiyama, Andrew J. Potter, Lindsay M. Sabik, Grace G. Stehlin, Amal N. Trivedi, Fredric D. Wolinsky

**Affiliations:** ^1^ Department of Family Medicine, UNC-Chapel Hill School of Medicine, Chapel Hill, North Carolina, USA.; ^2^ Cecil G. Sheps Center for Health Services Research, UNC-Chapel Hill, Chapel Hill, North Carolina, USA.; ^3^ Department of Health Policy and Management, Gillings School of Public Health, UNC-Chapel Hill, Chapel Hill, North Carolina, USA.; ^4^ Department of Political Science and Criminal Justice, California State University, Chico, California, USA.; ^5^ Department of Health Policy and Management, Graduate School of Public Health, University of Pittsburgh, Pittsburgh, Pennsylvania, USA.; ^6^ Department of Health Services, Policy and Practice, School of Public Health, Brown University, Providence, Rhode Island, USA.; ^7^ Department of Health Management and Policy, College of Public Health, University of Iowa, Iowa City, Iowa, USA.

**Keywords:** health centers, dual eligibles, disparities, hospital care

## Abstract

**Introduction:**

Health center use may reduce hospital-based care among Medicare–Medicaid dual eligibles, but racial and ethnic disparities in this population have not been widely studied. We examined the extent of racial and ethnic disparities in hospital-based care among duals using health centers and the degree to which disparities occur within or between health centers.

**Methods:**

We used 2012–2018 Medicare claims and health center data to model emergency department (ED) visits, observation stays, hospitalizations, and 30-day unplanned returns as a function of race and ethnicity among dual eligibles using health centers.

**Results:**

In rural and urban counties, age-eligible Black individuals had more ED visits (7.9 [4.0, 11.7] and 13.7 [10.0, 17.4] per 100 person-years) and were more likely to experience an unplanned return (1.4 [0.4, 2.4] and 1 [0.4, 1.6] percentage points [pp]) than White individuals, but were less likely to be hospitalized (−3.3 [−3.9, −2.8] and −1.2 [−1.6, −0.9] pp). In urban counties, age-eligible Black individuals were 1.2 [0.9, 1.5] pp more likely than White individuals to have observation stays. Other racial and ethnic groups used the same or less hospital-based care than White individuals. Including state and health center fixed effects eliminated Black versus White disparities in all outcomes, except hospitalization. Results were similar among disability–eligible duals.

**Conclusion:**

Racial and ethnic disparities in hospital-based care among dual eligibles are less common within than between health centers. If health centers are to play a more central role in eliminating racial and ethnic health disparities, these differences across health centers must be understood and addressed.

## Introduction

Limited primary care access often results in costly and potentially preventable hospital-based care use.^1–16^ Rates of hospital-based care are particularly high among the 12.3 million dual eligibles enrolled in both Medicare and Medicaid,^5,17^ and disparities in such care exist by race and ethnicity,^1–4,9,18^ socioeconomic status,^6,7^ and rurality.^19^

Federally qualified health centers (FQHCs) provide primary care to 30 million patients in underserved communities nationwide.^20^ FQHCs may reduce racial and ethnic disparities in health care access and quality,^21–24^ including hospital-based care,^25–32^ although findings remain mixed.^33–35^ Moreover, FQHCs served three times as many dual eligibles in 2018 versus 2005,^36,37^ and the proportion of dual eligibles is nearly seven times higher at FQHCs than among private physician practices.^36^

While FQHC use is associated with lower rates of hospital-based care among dual eligibles,^32^ the extent of racial and ethnic disparities in this population is unknown. Thus, we examined the extent of racial and ethnic disparities in hospital-based care among dual eligibles using FQHCs. Reasons for FQHCs' ability to reduce disparities are also unclear. FQHCs differ from other primary care providers in their provision of nonclinical enabling services to increase access to care, their focus on caring for low-income publicly insured and uninsured patients, and their patient majority governance structure, any or all of which might facilitate FQHCs' ability to reduce disparities.^38–41^ Yet, each FQHC tailors its services to meet particular community needs. Thus, we also examined the extent to which disparities in hospital-based care occur within or between FQHCs.

Based on racial and ethnic disparities in hospital quality,^42,43^ we hypothesized that racial and ethnic disparities would exist among FQHC users but would be more pronounced between—rather than within—FQHCs. Our findings document FQHC performance among a fast-growing population of interest to policymakers and inform the targeting of future resources, policies, and programs aiming to eliminate disparities within the health center program.

## Methods

### Data and sample

We used 2012–2018 inpatient and outpatient Medicare claims and enrollment data for 100% of dual eligibles enrolled in fee-for-service Medicare to define our sample, identify both hospital-based care and outpatient evaluation and management (E&M) visits, and adjust for an extensive set of patient characteristics. We limited our sample to full-benefit dual enrollees with 12 months of coverage during the year who received the plurality of their primary care in an FQHC. To reduce heterogeneity and ensure equal measurement periods, we excluded individuals who did not use primary care, had end-stage renal disease, or died during the year. We also required individuals to live in a primary care service area with an FQHC to ensure geographic access to a health center.^44^ While all 50 states and the District of Columbia are included in our study, only FQHCs that provided care to one or more dual eligible patients in our sample are included.

To identify FQHC users, we first identified all primary care visits before calculating the proportion of each individual's visits occurring in office-based, hospital-based, FQHC, rural health clinic, and other settings. We used type of bill and place of service codes in outpatient claims to identify facility bills for FQHCs and rural health clinics. Then, we used outpatient and carrier claims to identify all other primary care providers using specialty, current procedural terminology, and place of service codes.

Primary care visits included outpatient E&M visits to physicians (family practice, general practice, geriatrics, internal medicine), nurse practitioners, certified clinical nurse specialists, and physician assistants in office settings (99201–99215), nursing facilities (99304–99340), or patients' homes (99341–99350). We defined enrollees as FQHC users if a plurality of their primary care visits occurred in an FQHC versus the other four settings.^31,32,45^ As a robustness check, we used increasingly restrictive thresholds (i.e., >50%, ≥75%, and 100% of primary care visits) to define FQHC users.^32,46,47^

**FIG. 1.**
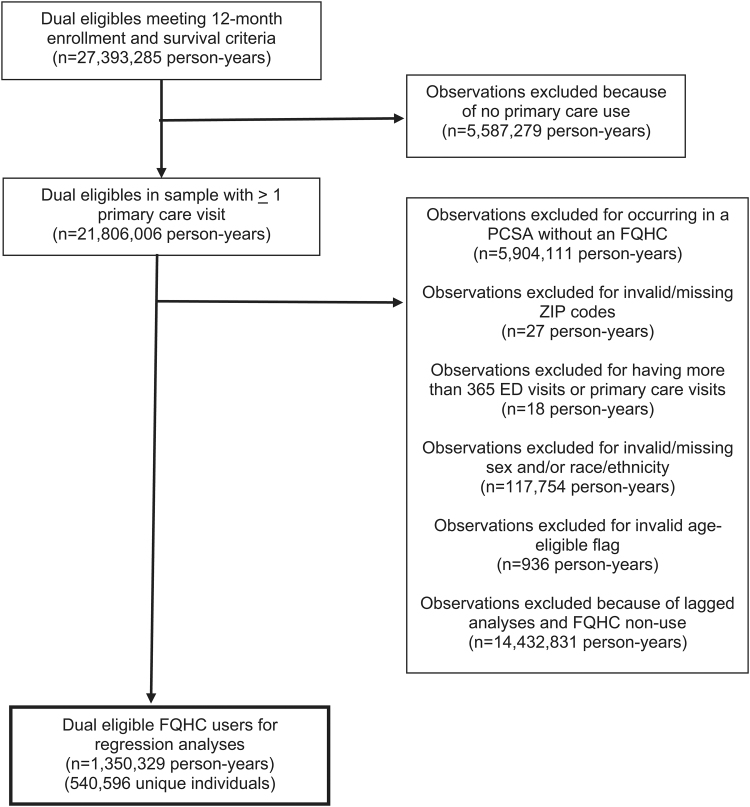
Sample selection flowchart. ED, emergency department; FQHC, federally qualified health center; PCSA, primary care service area.

We used National Provider Identifiers and the Uniform Data System^48^ to exclude FQHC “look-alikes” (i.e., providers meeting FQHC statutory requirements but not receiving federal funds) and attribute each FQHC visit to a specific grantee. We assigned individuals to their most frequently visited FQHC each year. Individuals who visited multiple FQHCs equally were assigned to the last FQHC they visited that year. These 1930 cases accounted for just 0.14% of our person-year sample. Finally, we lagged FQHC use by 1 year to ensure it preceded our hospital-based care outcomes. Thus, all individuals were in our data for at least 2 consecutive years. Figure 1 depicts sample selection.

### Outcomes

Given recent health care trends, we defined hospital-based care as not only emergency department (ED) visits and hospitalizations, but also observation stays and 30-day unplanned returns.^49^ We identified ED visits using revenue center codes (RCCs) 450, 451, 452, 456, and 459 or Healthcare Common Procedure Coding System (HCPCS) codes 99281–99285 in inpatient and outpatient claims,^50^ observation stays using RCCs 760 or 762 and HCPCS codes G0378 or G0379 in outpatient claims,^51,52^ and hospitalizations using inpatient claims. We defined unplanned returns as any ED, observation, or readmission within 30 days of discharge.^53^ The 30 days could cross calendar years with returns attributed to the year in which they happened, and need not occur at the same hospital, but we excluded index events after December 1, 2018 to ensure full follow-up. Based on event frequency, we defined ED visits as a count and other outcomes as binary indicators.

### Analytic approach

We estimated the extent of racial and ethnic disparities in hospital-based care among FQHC users, using a series of linear regression models:

(1) $$Outcome_{it}=\alpha_{0}+\beta_{1}Race_{i}+\beta_{2}X_{it}+\beta_{3}Year_{t}+\varepsilon_{it}$$

Where $Outcome_{it}$ is one of our hospital-based care outcomes for enrollee *i* in year *t*. Our key independent variables are a construct of race and ethnicity indicators ($Race_{i}$), defined using RTI race codes, which improve Hispanic and Asian/Pacific Islander identification.^54^ $X_{it}$ is a vector of enrollee characteristics, including age, sex, number of annual primary care visits, count of chronic conditions, and—for age-eligible duals—an indicator for originally being Medicare eligible based on disability. $Year_{t}$is a vector of binary indicators to capture secular time trends.

Next, we repeated these analyses, including state fixed effects ($Stat{e_{j}}^{}$) to adjust for unobserved time-invariant factors (e.g., practice patterns, socioeconomic status, health care infrastructure and workforce, Medicaid payment policy) potentially associated with our outcomes.^55^ For instance, FQHCs in Medicaid expansion states have benefited financially while FQHCs in nonexpansion states have not,^56^ leading to improvements in chronic disease care and reduced disparities between Black and White FQHC patients with hypertension.^57^

(2) $$Outcome_{ijt}=\alpha_{0}+\beta_{1}Race_{i}+\beta_{2}X_{it}+\beta_{3}Year_{t}+\beta_{4}State_{j}+\varepsilon_{ijt}$$

Then, we included fixed effects for each unique state and FQHC grantee combination ($StateFQHC_{j})$, allowing us to identify the extent of racial and ethnic disparities on average *within* a given FQHC.

(3) $$Outcome_{ijt}=\alpha_{0}+\beta_{1}Race_{i}+\beta_{2}X_{it}+\beta_{3}Year_{t}+\beta_{4}StateFQHC_{j}+\varepsilon_{ijt}$$

Finally, we ran sensitivity analyses on our Equation 3 models. First, because long-term care residents have higher rates of hospital-based care,^58,59^ which may be more closely related to nursing home rather than primary care quality, we excluded 42,695 observations for individuals who received nursing facility care during the year. Then, we used an increasingly restrictive threshold for FQHC user assignment. To account for multiple comparisons, we employed a Bonferroni correction, which reduced our alpha level from 0.05 to 0.01. Because dual eligibles are heterogeneous and FQHCs may operate differently in rural versus urban areas, we stratified all models by current reason for eligibility and rurality and used person-level robust-clustered standard errors to account for multiple observations of individuals over time. The University of North Carolina at Chapel Hill Institutional Review Board approved this study.

## Results

### Summary statistics

Summary statistics appear in Table 1, stratified by current basis for eligibility and rurality. Age-eligible duals are obviously older than disability-eligible duals, but they are also less likely to be male or White and more likely to be Hispanic than disability-eligible duals. While they use comparable levels of outpatient care, age-eligible duals have more chronic conditions and use more hospital-based care than disability-eligible duals. Within eligibility groups, rural residents are slightly older, more likely to be White, and have marginally more chronic conditions. They visit primary care providers slightly more and specialists slightly less than urban residents. Finally, hospital-based care is somewhat higher among age-eligible duals in rural versus urban areas, but somewhat lower among disability-eligible duals in rural versus urban areas.

**Table 1.** Descriptive Statistics for Federally Qualified Health Center Users in Regression Analyses

	Age-eligible duals		Disability-eligible duals	
	Rural	Urban	Rural	Urban
Mean age	75.2 [IQR: 69, 80]	74.2 [IQR: 69, 78]	49.5 [IQR: 42, 58]	49.4 [IQR: 42, 58]
% Male	31.7 [31.5, 32]	36.4 [36.2, 36.5]	44.5 [44.3, 44.8]	45.7 [45.5, 45.8]
Race and ethnicity				
% White	63.5 [63.3, 63.8]	32.0 [31.9, 32.1]	74.4 [74.2, 74.6]	50.9 [50.8, 51.0]
% Black	19.0 [18.8, 19.2]	17.3 [17.2, 17.4]	16.0 [15.8, 16.2]	26.2 [26, 26.3]
% Asian/Pacific Islander	0.8 [0.7, 0.8]	15.5 [15.4, 15.6]	0.6 [0.6, 0.7]	2.6 [2.6, 2.6]
% American Indian/Alaska Native	2.5 [2.4, 2.6]	0.5 [0.5, 0.6]	2.1 [2, 2.2]	0.9 [0.8, 0.9]
% Hispanic	13.7 [13.5, 13.8]	33.9 [33.8, 34]	6.4 [6.3, 6.5]	18.8 [18.7, 18.9]
% Other	0.5 [0.5, 0.6]	0.8 [0.8, 0.8]	0.5 [0.4, 0.5]	0.6 [0.6, 0.6]
Health status				
Mean no. of chronic conditions	4.8 [IQR: 3, 7]	4.3 [IQR: 2, 6]	3.0 [IQR: 1, 4]	2.9 [IQR: 1, 4]
% Old with disabilities	33.6 [33.4, 33.9]	24.9 [24.8, 25]	N/A	N/A
Health use				
Mean total E&M visits with PCP	10.1 [IQR: 5, 13]	9.7 [IQR: 4, 12]	10.3 [IQR: 4, 14]	9.8 [IQR: 4, 13]
Mean total E&M visits with specialist	3.3 [IQR: 0, 5]	3.8 [IQR: 0, 5]	3.6 [IQR: 0, 5]	4.3 [IQR: 0, 6]
Hospital-based care				
Mean total ED visits	1.1 [IQR: 0, 1]	0.9 [IQR: 0, 1]	1.5 [IQR: 0, 2]	1.6 [IQR: 0, 2]
% at least one observation stay	9.4 [9.2, 9.5]	7.7 [7.6, 7.8]	6.4 [6.3, 6.6]	7.2 [7.1, 7.2]
% at least one inpatient stay	21.4 [21.1, 21.6]	18.0 [17.9, 18.1]	16.7 [16.5, 16.9]	18.3 [18.2, 18.4]
% at least one unplanned return	31.0 [30.6, 31.3]	27.8 [27.6, 28]	34.3 [34, 34.7]	35.8 [35.6, 35.9]
	*N*=135,336	*N*=502,879	*N*=152,472	*N*=559,642

*Note:* Values in brackets are 95% confidence intervals, in less IQR is specified.

ED, emergency department; E&M, evaluation and management; IQR, interquartile range; N/A, not applicable; PCP, primary care provider.

### Racial and ethnic disparities in hospital-based care among dual-eligible FQHC users

Table 2 presents marginal effects of our race indicators from our model without state and FQHC fixed effects. These results demonstrate patterns of disparities among all dual eligibles who rely on FQHCs for primary care. Among age-eligible duals, Black individuals have a higher ED visit rate than White individuals. In rural counties, Black individuals averaged 7.9 more ED visits per 100 person-years than White individuals (a 7.2% increase relative to the baseline ED visit rate in our sample), and in urban counties, they averaged 13.7 more ED visits per 100 person-years than White individuals (a 15.2% increase relative to baseline). This also translated into Black individuals having an increased probability of experiencing a 30-day unplanned return compared with White individuals (a 1.4 percentage point [pp] increase in rural counties and 1 pp increase in urban counties). In urban counties, age-eligible duals who are Black are 1.2 pp more likely to have an observation stay during the year than those who are White. By contrast, Black individuals in rural counties were 3.3 pp less likely to be hospitalized than White individuals, while Black individuals in urban counties were 1.2 pp less like likely to be hospitalized than their White peers.

**Table 2.** Marginal Effects of Race and Ethnicity from Regression Models Without Fixed Effects

Outcome	Black		Asian/Pacific Islander		Hispanic		American Indian/Alaska Native		Other	
	Rural	Urban	Rural	Urban	Rural	Urban	Rural	Urban	Rural	Urban
Age-eligible adults										
ED visits	7.9^***^ [4.0, 11.7]	13.7^***^ [10.0, 17.4]	−2.8 [−12.8, 7.2]	−41.2^***^ [−43.1, −39.3]	−14.0^***^ [−17.7, −10.2]	−23.9^***^ [−25.7, −22.1]	−0.3 [−9.8, 9.3]	25.5^***^ [12.8, 38.2]	−13.4 [−26.0, −0.7]	−23.9^***^ [−30.9, −16.9]
Observation	−0.6 [−1.0, −0.1]	1.2^***^ [0.9, 1.5]	−1.1 [−2.5, 0.3]	−3.0^***^ [−3.2, −2.7]	−2.7^***^ [−3.2, −2.3]	−2.1^***^ [−2.3, −1.9]	0.0 [−1.1, 1.1]	1.3 [0.1, 2.5]	1.2 [−0.9, 3.3]	−0.4 [−1.3, 0.5]
Inpatient	−3.3^***^ [−3.9, −2.8]	−1.2^***^ [−1.6, −0.9]	0.8 [−1.3, 2.9]	−5.0^***^ [−5.3, −4.7]	−4.8^***^ [−5.4, −4.1]	−5.7^***^ [−5.9, −5.4]	4.1^***^ [2.7, 5.6]	1.3 [−0.2, 2.8]	−3.2 [−6.0, −0.5]	−4.1^***^ [−5.2, −2.9]
Unplanned return	1.4^*^ [0.4, 2.4]	1.0^*^ [0.4, 1.6]	1.6 [−2.9, 6.1]	−7.8^***^ [−8.5, −7.1]	−2.4^***^ [−3.5, −1.2]	−4.1^***^ [−4.6, −3.6]	0.3 [−2.2, 2.8]	3.9^*^ [1.1, 6.7]	−0.8 [−5.9, 4.3]	−4.7^**^ [−7.1, −2.2]
Disability-eligible adults										
ED visits	12.9^***^ [6.5, 19.3]	12.6^***^ [8.8, 16.4]	−17.5 [−36.8, 1.7]	−56.2^***^ [−62.4, −50.0]	−9.0 [−16.8, −1.2]	−31.3^***^ [−34.7, −27.9]	8.1 [−6.5, 22.6]	30.7^***^ [16.1, 45.4]	9.1 [−28.4, 46.6]	−30.9^**^ [−47.7, −14.1]
Observation	−0.6^*^ [−1.0, −0.2]	0.7^***^ [0.5, 0.9]	−1.5 [−2.8, −0.1]	−2.0^***^ [−2.3, −1.6]	−0.9^**^ [−1.4, −0.4]	−1.2^***^ [−1.4, −1.1]	1.1 [0.2, 2.1]	0.8 [0.0, 1.6]	−0.9 [−2.9, 1.0]	−0.3 [−1.3, 0.6]
Inpatient	−2.0^***^ [−2.5, −1.4]	−0.9^***^ [−1.2, −0.6]	−0.4 [−2.6, 1.7]	−3.8^***^ [−4.4, −3.1]	−1.7^***^ [−2.5, −0.9]	−4.1^***^ [−4.4, −3.8]	3.5^***^ [2.0, 5.0]	1.6 [0.4, 2.9]	1.1 [−2.0, 4.2]	−2.2^*^ [−3.5, −0.8]
Unplanned return	0.5 [−0.5, 1.5]	−0.2 [−0.6, 0.3]	−6.1^*^ [−10.7, −1.5]	−7.9^***^ [−9.3, −6.5]	−0.8 [−2.3, 0.8]	−4.2^***^ [−4.7, −3.7]	1.9 [−0.8, 4.6]	4.3^***^ [2.2, 6.3]	−1.9 [−7.4, 3.6]	−4.1^*^ [−6.7, −1.6]

*Note:* ED visit coefficients indicate magnitude per 100 person-years and all other coefficients indicate percentage point change. All coefficients are interpreted relative to the White racial category.

^*^ *p*<0.01, ^**^p<0.001, ^***^p<0.0001.

Conversely, the associations between other racial and ethnic categories and our outcomes were generally negative or insignificant, indicating these racial and ethnic patient groups use the same or less hospital-based care than similarly situated White individuals. American Indian/Alaska Native individuals were the exception. Regardless of their basis for eligibility, American Indian/Alaska Native individuals in rural counties were more likely to be hospitalized than White individuals, while those in urban counties had more ED visits and more unplanned returns. While there were some differences in magnitude, we observed a comparable pattern of results among disability-eligible duals regardless of race or ethnicity.

### State fixed effects models

Table 3 presents marginal effects of our race and ethnicity indicators from our model with state fixed effects. This change in model specification moved some marginal effects upward (i.e., strengthening positive marginal effects and weakening negative ones) and some downward (i.e., weakening positive marginal effects and strengthening negative ones), while only a few results became statistically significant. Overall patterns of racial and ethnic disparities in hospital-based care among dual eligibles who use FQHCs persisted, however. For instance, among age-eligible duals, Black individuals now have 11.2 more ED visits per 100 person-years than White individuals in rural counties, while in urban counties Black individuals have 15 more ED visits per 100 person-years than White individuals. Meanwhile, Black individuals remained less likely than White individuals to be hospitalized, but the effects were smaller (e.g., a 2.7 pp reduction among age-eligible duals in rural counties and a 1.2 pp reduction in urban counties).

**Table 3.** Marginal Effects of Race and Ethnicity from Regression Models with State Fixed Effects

Outcome	Black		Asian/Pacific Islander		Hispanic		American Indian/Alaska Native		Other	
	Rural	Urban	Rural	Urban	Rural	Urban	Rural	Urban	Rural	Urban
Age-eligible adults										
ED visits	11.2^***^ [5.9, 16.5]	15.0^***^ [10.5, 19.5]	−13.2 [−24.3, −2.2]	−39.5^***^ [−41.4, −37.5]	−11.4^***^ [−16.5, −6.2]	−20.7^***^ [−22.7, −18.7]	4.3 [−7.6, 16.1]	21.5^*^ [8.6, 34.4]	−17.1^*^ [−29.8, −4.4]	−24.3^***^ [−31.2, −17.3]
Observation	−0.7 [−1.3, −0.0]	0.7^***^ [0.4, 1.0]	−0.1 [−1.6, 1.3]	−1.6^***^ [−1.8, −1.4]	−1.6^***^ [−2.2, −1.0]	−1.1^***^ [−1.3, −0.8]	1.1 [−0.3, 2.4]	0.7 [−0.5, 1.9]	1.6 [−0.5, 3.7]	0.0 [−0.9, 0.9]
Inpatient	−2.7^***^ [−3.5, −1.9]	−1.2^***^ [−1.5, −0.8]	−0.7 [−3.0, 1.5]	−4.9^***^ [−5.2, −4.5]	−4.3^***^ [−5.2, −3.5]	−5.3^***^ [−5.6, −5.0]	1.1 [−0.6, 2.8]	−0.3 [−1.8, 1.3]	−3.7^*^ [−6.4, −1.0]	−4.2^***^ [−5.4, −3.0]
Unplanned return	2.9^***^ [1.6, 4.3]	1.3^***^ [0.7, 1.9]	−1.2 [−5.9, 3.5]	−7.7^***^ [−8.4, −6.9]	−1.4 [−3.0, 0.1]	−3.6^***^ [−4.2, −3.1]	0.8 [−2.1, 3.7]	3.2 [0.4, 6.1]	−0.8 [−5.9, 4.4]	−4.7^**^ [−7.2, −2.3]
Disability-eligible adults										
ED visits	13.4^*^ [5.2, 21.6]	13.9^***^ [9.9, 17.9]	−28.9^*^ [−47.8, −9.9]	−59.0^***^ [−65.3, −52.8]	−17.1^**^ [−26.1, −8.2]	−32.7^***^ [−36.3, −29.0]	9.6 [−5.6, 24.9]	25.4^**^ [10.7, 40.1]	3.7 [−33.9, 41.3]	−32.8^**^ [−49.7, −16.0]
Observation	−0.5 [−1.0, −0.0]	0.6^***^ [0.4, 0.8]	−0.9 [−2.2, 0.5]	−1.5^***^ [−1.9, −1.1]	−1.3^***^ [−1.9, −0.7]	−1.0^***^ [−1.2, −0.8]	1.3 [0.3, 2.3]	0.7 [−0.1, 1.5]	−0.7 [−2.6, 1.2]	−0.3 [−1.3, 0.6]
Inpatient	−1.3^**^ [−2.1, −0.6]	−0.9^***^ [−1.2, −0.6]	−2.1 [−4.3, 0.1]	−4.4^***^ [−5.0, −3.8]	−2.8^***^ [−3.8, −1.8]	−4.7^***^ [−5.0, −4.4]	2.5^*^ [0.9, 4.1]	0.9 [−0.3, 2.2]	0.7 [−2.4, 3.8]	−2.7^***^ [−4.0, −1.4]
Unplanned return	0.6 [−0.8, 1.9]	0.3 [−0.2, 0.7]	−7.8^*^ [−12.6, −3.0]	−8.6^***^ [−10.1, −7.2]	−1.8 [−3.5, 0.0]	−4.8^***^ [−5.3, −4.2]	1.5 [−1.3, 4.4]	3.3^*^ [1.2, 5.4]	−2.7 [−8.2, 2.8]	−4.4^**^ [−7.0, −1.8]

*Note:* ED visit coefficients indicate magnitude per 100 person-years and all other coefficients indicate percentage point change. All coefficients are interpreted relative to the White racial category.

^*^ *p*<0.01, ^**^p<0.001, ^***^p<0.0001.

Most importantly, the magnitudes of these marginal effects (vs. models without state fixed effects) change enough to confirm the importance of adjusting for state, although this adjustment does not change the conclusion that racial and ethnic disparities in hospital-based care exist among dual eligibles FQHC users.

### State and FQHC grantee fixed effects models

Table 4 presents marginal effects of our race and ethnicity indicators from our model with state and FQHC grantee fixed effects. These fully adjusted models indicate the extent of racial and ethnic disparities among patients who reside in the same state and seek care at the same FQHC. We no longer see differences between Black and White individuals' probability of experiencing an observation stay or unplanned return. Additionally, the difference in ED visits between Black and White individuals is no longer significant, except for age-eligible Black duals in urban areas who continue to have 9.7 more ED visits per 100 person-years than White duals. Black versus White disparities in the probability of hospitalization persisted, although the gap narrowed slightly among rural residents and widened slightly among urban residents.

**Table 4.** Marginal Effects of Race and Ethnicity from Regression Models with State and Federally Qualified Health Center Grantee Fixed Effects

Outcome	Black		Asian/Pacific Islander		Hispanic		American Indian/Alaska Native		Other	
	Rural	Urban	Rural	Urban	Rural	Urban	Rural	Urban	Rural	Urban
Age-eligible adults										
ED visits	7.8 [1.6, 14.0]	9.7^***^ [5.4, 14.0]	−14.7 [−26.7, −2.7]	−32.7^***^ [−35.4, −30.0]	−5.8 [−11.6, −0.0]	−16.7^***^ [−19.0, −14.3]	13.8 [0.2, 27.4]	17.2 [2.3, 32.1]	−11.8 [−24.9, 1.3]	−22.0^***^ [−29.2, −14.8]
Observation	−0.2 [−0.9, 0.5]	0.4 [0.0, 0.7]	−0.2 [−1.7, 1.3]	−1.5^***^ [−1.8, −1.2]	−1.1^*^ [−1.8, −0.4]	−0.9^***^ [−1.1, −0.6]	0.3 [−1.3, 1.8]	0.5 [−0.8, 1.8]	0.5 [−1.7, 2.6]	0.0 [−0.9, 1.0]
Inpatient	−3.0^***^ [−3.9, −2.0]	−1.9^***^ [−2.3, −1.4]	−0.3 [−2.6, 2.0]	−4.4^***^ [−4.8, −3.9]	−3.1^***^ [−4.1, −2.2]	−4.4^***^ [−4.7, −4.1]	1.2 [−0.7, 3.1]	−1.1 [−2.8, 0.6]	−2.8 [−5.6, −0.1]	−4.1^***^ [−5.3, −2.9]
Unplanned return	1.8 [0.2, 3.4]	0.6 [−0.2, 1.3]	−0.0 [−5.1, 5.0]	−6.5^***^ [−7.5, −5.6]	−0.6 [−2.4, 1.2]	−3.1^***^ [−3.7, −2.5]	1.6 [−1.7, 4.8]	3.2 [0.1, 6.3]	−0.0 [−5.3, 5.3]	−4.0^*^ [−6.4, −1.5]
Disability-eligible adults										
ED visits	11.8 [2.6, 21.1]	4.2 [−0.2, 8.6]	−26.6^*^ [−46.4, −6.9]	−59.0^***^ [−67.2, −50.8]	−8.7 [−18.7, 1.2]	−28.7^***^ [−33.1, −24.3]	11.9 [−4.4, 28.2]	20.2^*^ [5.1, 35.3]	2.5 [−32.7, 37.6]	−35.0^***^ [−51.9, −18.1]
Observation	0.0 [−0.5, 0.6]	0.2 [−0.0, 0.4]	−1.1 [−2.4, 0.3]	−1.6^***^ [−2.0, −1.1]	−0.8 [−1.5, −0.2]	−0.9^***^ [−1.1, −0.7]	0.4 [−0.6, 1.5]	0.7 [−0.1, 1.6]	−0.8 [−2.6, 1.1]	−0.6 [−1.6, 0.3]
Inpatient	−1.7^***^ [−2.5, −0.8]	−1.9^***^ [−2.2, −1.6]	−2.1 [−4.3, 0.1]	−4.8^***^ [−5.6, −4.1]	−2.2^***^ [−3.2, −1.2]	−4.4^***^ [−4.8, −4.1]	2.4^*^ [0.7, 4.0]	0.7 [−0.6, 2.0]	0.8 [−2.4, 3.9]	−3.2^***^ [−4.5, −1.8]
Unplanned return	0.6 [−0.9, 2.1]	−0.6 [−1.2, −0.1]	−7.7^*^ [−12.7, −2.6]	−8.5^***^ [−10.0, −7.0]	−1.3 [−3.2, 0.6]	−4.1^***^ [−4.8, −3.5]	1.4 [−1.6, 4.4]	2.1 [−0.1, 4.2]	−2.7 [−8.2, 2.8]	−4.5^**^ [−7.1, −1.9]

*Note:* ED visit coefficients indicate magnitude per 100 person-years and all other coefficients indicate percentage point change. All coefficients are interpreted relative to the White racial category.

^*^ *p*<0.01, ^**^p<0.001, ^***^p<0.0001.

Similarly, among age-eligible duals, no significant differences in outcomes remained between American Indian/Alaska Native and White individuals regardless of rural or urban residence, nor were there any significant differences between Asian/Pacific Islander and White individuals in rural areas. Among disability-eligible duals, American Indian/Alaska Native individuals in urban counties still had more ED visits than White individuals (20.2 more ED visits per 100 person-years) and those in rural counties were 2.4 pp more likely than White individuals to be hospitalized.

Other significant associations between race and ethnicity and our outcomes persisted, but in most cases disparities between these groups and White patients were narrowed. In fact, the only gaps that widened were for disability-eligible duals in urban counties and were specific to observation stays and hospitalization among Asian/Pacific Islander individuals, and ED visits, hospitalizations, and unplanned returns among those in the “Other” race group.

Our sensitivity analyses, excluding nursing facility users (Supplementary Appendix Table SA1) resulted in modest changes in the magnitude of coefficients, but overall trends remained consistent. However, using an increasingly restrictive threshold for FQHC user assignment (Supplementary Appendix Tables SA2 and SA3) resulted in some reductions in racial and ethnic disparities in hospital-based care as dual eligibles received a greater proportion of their primary care at an FQHC.

## Discussion

In this study, we found racial and ethnic disparities in hospital-based care among dual eligibles receiving a plurality of their primary care at FQHCs. Overall, we observed that: (1) Black individuals had a higher rate of ED visits than White individuals, but a lower probability of hospitalization; (2) American Indian/Alaska Native individuals in rural counties were more likely to be hospitalized, while those in urban counties had a higher rate of ED visits than White individuals; and (3) Individuals in the remaining racial and ethnic groups used less hospital-based care than White individuals. We also found that adjusting for state-level differences noticeably changed the magnitude of our estimates, but not the overall pattern of our findings. Perhaps most importantly, we found disparities were generally attenuated, and in some cases eliminated, in our fully adjusted models accounting for both state and FQHC fixed effects.

Our findings suggest that racial and ethnic disparities in hospital-based care among dual eligibles are less common within a given FQHC, but more common between FQHCs and across states. Prior studies have documented that FQHCs are better equipped than other settings to provide access to high-quality primary care,^60^ and there is continued interest in leveraging FQHCs to combat health disparities in cancer care,^61–63^ viral hepatitis screening,^64,65^ prenatal care,^66^ oral health,^67^ and COVID-19 vaccination.^68^ Thus, our findings are important because they underscore that significant racial and ethnic disparities exist between FQHCs within the health center program, even after adjusting for state-level differences like Medicaid expansion^57,69–71^ and access to telehealth^72^ that partially explain these disparities. If FQHCs are to play a more central role in eliminating racial and ethnic health disparities, these differences across FQHCs must be understood and addressed.

While identifying specific mechanisms is beyond the scope of this study, we offer some potential explanations for our findings. First, differences in FQHC service provision may lead to disparate outcomes between FQHC patients. For example, serious mental illness often exacerbates health disparities.^65,73^ Patients at FQHCs that provide integrated primary care and behavioral health services may experience better outcomes,^74,75^ whereas patients at FQHCs without such integrated services may experience worse outcomes. Our findings support a scenario in which all patients at a given FQHC either have or do not have access to such integrated services regardless of their race or ethnicity, but racially and ethnically minoritized patients are disproportionately served by FQHCs lacking such services. Variation in the social and environmental conditions of different FQHC service areas may also explain our findings. For example, if communities with a high proportion of racially and ethnically minoritized individuals also have limited access to pharmacies or healthy food, residents there will face additional obstacles to maintaining their health and avoiding hospital-based care.

Moreover, even in our fully adjusted models, some racial and ethnic disparities in hospital-based care persist. These likely arise from deeply entrenched structural and interpersonal racism that harm racially and ethnically minoritized individuals in ways receiving care at an FQHC can only partially address.^76–79^ In fact, after controlling for political leanings, socioeconomic conditions, and health status, states with a higher degree of implicit racial bias have fewer FQHC delivery sites.^80^ Notably, some disparities we identify arise from White patients having seemingly “worse” outcomes (i.e., more ED visits, observation stays, and hospitalizations) than patients in the Asian, Hispanic, and Other racial and ethnic groups. While we generally consider reductions in hospital-based care to be desirable, we cannot definitively conclude from claims data whether a given visit was preventable, and not receiving needed hospital-based care is undesirable. In short, both overuse and underuse of hospital-based care should be minimized.

This study has limitations. First, the observational design precludes us from making causal inferences. Second, our data are limited to dual eligibles with fee-for-service Medicare. Thus, our findings may not generalize to other populations, including Medicare-managed care enrollees. Finally, to ensure full person-year observations, we excluded individuals who died during the year. To the extent our resulting sample is healthier, this may also reduce generalizability.

Most extant studies have focused on the extent to which FQHCs reduce racial and ethnic disparities in primary care access and quality relative to other primary care settings. In contrast, our study is the first we know of to examine the extent of racial and ethnic disparities in hospital-based care among dual eligible FQHC users, and the first to examine the extent to which these disparities occur primarily between versus within FQHCs. We find substantial evidence of racial and ethnic disparities in hospital-based care within the health center program, but these disparities are more likely to result from differences between FQHCs, rather than differences within any given FQHC. Still, some disparities also persist within FQHCs, and may reflect both underuse and overuse of hospital-based care.

Future research should seek to identify the factors driving these disparities within and between FQHCs. Once identified, policymakers and practitioners can employ evidence-based strategies to reduce high rates of hospital-based care at underperforming FQHCs and invest in FQHCs to reduce and eliminate disparities, particularly among a high-need, high-cost population like dual eligibles.

## Supplementary Material

Supplemental data

## Abbreviations Used

E&M: evaluation and management
ED: emergency department
FQHC: federally qualified health center
HCPCS: Healthcare Common Procedure Coding System
IQR: interquartile range
PCP: primary care provider
PCSA: primary care service area
pp: percentage point
RCC: revenue center code
