# Supplementary material for: Racial and Ethnic Disparities in Hospital-Based Care Among Dual Eligibles Who Use Health Centers
Source: Health Equity. 2023 Jan 13;7(1):9–18. doi: 10.1089/heq.2022.0037 (PMC9892926; doi:10.1089/heq.2022.0037)
Supplement: Supplemental data [file Supp_AppendixS1-S3.docx]

**Appendix Table 1. Marginal Effects of Race and Ethnicity from Regression Models with State and FQHC Grantee Fixed Effects, Excluding Nursing Facility Users**

|  | **Age-Eligible Adults** | | | | | | | | | |
| --- | --- | --- | --- | --- | --- | --- | --- | --- | --- | --- |
|  | **Black** | | **Asian/PI** | | **Hispanic** | | **AI/AN** | | **Other** | |
| **Outcome** | Rural | Urban | Rural | Urban | Rural | Urban | Rural | Urban | Rural | Urban |
| ED Visits | 8.2*  [2.0, 14.4] | 11.6***  [7.5, 15.7] | -13.9  [-25.9, -1.9] | -28.2***  [-30.7, -25.7] | -5.2  [-10.9, 0.5] | -11.7***  [-13.8, -9.6] | 14.3  [1.5, 27.2] | 18.8  [3.8, 33.8] | -10.7  [-23.5, 2.1] | -18.3***  [-24.9, -11.7] |
| Observation | -0.3  [-1.0, 0.5] | 0.6**  [0.2, 0.9] | -0.2  [-1.7, 1.3] | -1.1***  [-1.4, -0.8] | -1.1*  [-1.7, -0.4] | -0.5**  [-0.7, -0.2] | 0.6  [-1.0, 2.1] | 0.5  [-0.7, 1.8] | 0.9  [-1.2, 3.1] | 0.1  [-0.8, 1.0] |
| Inpatient | -2.9***  [-3.8, -2.0] | -1.4***  [-1.8, -1.0] | -0.3  [-2.6, 2.0] | -3.1***  [-3.6, -2.7] | -2.6***  [-3.6, -1.7] | -3.1***  [-3.4, -2.8] | 1.4  [-0.5, 3.3] | -0.5  [-2.1, 1.2] | -2.6  [-5.3, 0.1] | -3.3***  [-4.4, -2.2] |
| Unplanned Return | 2.1  [0.5 - 3.8] | 0.7  [-0.1 - 1.4] | 0.1  [-5.2 - 5.3] | -6.0***  [-7.0 - -5.0] | -0.3  [-2.1 - 1.6] | -2.6***  [-3.2 - -1.9] | 1.8  [-1.5 - 5.2] | 3.9  [0.6 - 7.2] | 0.8  [-4.6 - 6.1] | -4.2*  [-6.7 - -1.6] |
|  | **Disability-Eligible Adults** | | | | | | | | | |
|  | **Black** | | **Asian/PI** | | **Hispanic** | | **AI/AN** | | **Other** | |
| **Outcome** | Rural | Urban | Rural | Urban | Rural | Urban | Rural | Urban | Rural | Urban |
| ED Visits | 11.9  [2.6, 21.1] | 5.6  [1.3, 9.9] | -27.3*  [-46.8, -7.8] | -56.1***  [-63.9, -48.4] | -8.5  [-18.3, 1.3] | -26.5***  [-30.8, -22.2] | 10.3  [-5.8, 26.3] | 20.3*  [5.2, 35.3] | 3.0  [-32.4, 38.4] | -32.6**  [-49.7, -15.5] |
| Observation | 0.0  [-0.5, 0.6] | 0.3  [0.1, 0.5] | -0.9  [-2.3, 0.4] | -1.5***  [-1.9, -1.0] | -0.8*  [-1.5, -0.2] | -0.7***  [-1.0, -0.5] | 0.3  [-0.7, 1.3] | 0.8  [-0.0, 1.6] | -0.6  [-2.5, 1.3] | -0.7  [-1.6, 0.2] |
| Inpatient | -1.7***  [-2.5, -0.8] | -1.7***  [-2.0, -1.4] | -1.9  [-4.1, 0.3] | -4.4***  [-5.2, -3.7] | -2.1***  [-3.1, -1.1] | -3.9***  [-4.2, -3.6] | 2.3*  [0.6, 3.9] | 0.7  [-0.6, 2.0] | 0.8  [-2.3, 4.0] | -2.9***  [-4.2, -1.6] |
| Unplanned Return | 0.7  [-0.9, 2.2] | -0.5  [-1.0, 0.1] | -7.3*  [-12.4, -2.2] | -8.4***  [-9.9, -6.8] | -1.2  [-3.1, 0.7] | -3.9***  [-4.6, -3.3] | 1.2  [-1.8, 4.2] | 1.7  [-0.5, 3.9] | -3.0  [-8.5, 2.6] | -4.4*  [-7.0, -1.7] |

*p<0.01, **p<0.001, ***p<0.0001

Note: ED visit coefficients indicate magnitude per 100 person-years and all other coefficients indicate percentage point change

**Appendix Table 2. Marginal Effects of Race and Ethnicity from Models with State and FQHC Grantee Fixed Effects Among Age-Eligible Adults, by Increasingly Restrictive FQHC User Thresholds**

|  | **Black** | | | **Asian/Pacific Islander** | | | **Hispanic** | | | **American Indian/Alaskan Native** | | | **Other Race** | | |
| --- | --- | --- | --- | --- | --- | --- | --- | --- | --- | --- | --- | --- | --- | --- | --- |
|  | **Rural** | | | | | | | | | | | | | | |
| **Outcome** | 50% | 75% | 100% | 50% | 75% | 100% | 50% | 75% | 100% | 50% | 75% | 100% | 50% | 75% | 100% |
| ED Visits | 8.2* | 7.8 | 7.8* | -14.3 | -15.1 | -16.0* | -5.8 | -6.2 | -8.9* | 14.4 | 15.2 | 12.8 | -11.2 | -7.4 | -4.2 |
| Observation | -0.2 | -0.1 | -0.1 | -0.4 | -0.1 | -0.9 | -1.1* | -0.9* | -1.2* | 0.4 | 0.7 | 1.1 | 0.5 | 1.0 | 0.9 |
| Inpatient | -2.9*** | -2.7*** | -2.6*** | -0.3 | 0.2 | -0.1 | -3.0*** | -3.1*** | -3.2*** | 1.1 | 1.2 | 1.1 | -2.9 | -2.9 | -1.9 |
| Unplanned Returns | 2.0 | 2.3* | 2.0 | 0.2 | 0.1 | -1.1 | -0.4 | -0.3 | -0.7 | 1.6 | 1.1 | 0.5 | 0.6 | 1.4 | 1.1 |
|  | **Urban** | | | | | | | | | | | | | | |
| **Outcome** | 50% | 75% | 100% | 50% | 75% | 100% | 50% | 75% | 100% | 50% | 75% | 100% | 50% | 75% | 100% |
| ED Visits | 9.9*** | 10.3*** | 7.8* | -32.6*** | -31.7*** | -16.0* | -16.6*** | -16.2*** | -8.9* | 18.0 | 16.9 | 12.8 | -22.1*** | -21.5*** | -4.2 |
| Observation | 0.4 | 0.5* | 0.5* | -1.4*** | -1.4*** | -1.3*** | -0.8*** | -0.8*** | -0.8*** | 0.6 | 1.0 | 0.5 | 0.1 | -0.2 | -0.1 |
| Inpatient | -1.8*** | -1.8*** | -1.6*** | -4.3*** | -4.2*** | -4.1*** | -4.4*** | -4.4*** | -4.4*** | -1.1 | -1.0 | -2.1 | -4.1*** | -4.3*** | -4.4*** |
| Unplanned Returns | 0.6 | 0.8 | 0.9 | -6.5*** | -6.5*** | -6.6*** | -3.1*** | -3.0*** | -2.9*** | 3.4 | 3.7 | 2.1 | -4.1* | -4.1* | -3.9* |

*p<0.01, **p<0.001, ***p<0.0001

Note: ED visit coefficients indicate magnitude per 100 person-years and all other coefficients indicate percentage point change

**Appendix Table 3. Marginal Effects of Race and Ethnicity from Models with State and FQHC Grantee Fixed Effects Among Disability-Eligible Adults, by Increasingly Restrictive FQHC User Thresholds**

|  | **Black** | | | **Asian/Pacific Islander** | | | **Hispanic** | | | **American Indian/Alaskan Native** | | | **Other Race** | | |
| --- | --- | --- | --- | --- | --- | --- | --- | --- | --- | --- | --- | --- | --- | --- | --- |
|  | **Rural** | | | | | | | | | | | | | | |
| **Outcome** | 50% | 75% | 100% | 50% | 75% | 100% | 50% | 75% | 100% | 50% | 75% | 100% | 50% | 75% | 100% |
| ED Visits | 14.0* | 13.0* | 7.7 | -27.3* | -24.5 | -28.5* | -8.1 | -7.3 | -6.0 | 13.6 | 10.1 | 3.0 | 4.1 | 10.2 | 9.7 |
| Observation | 0.1 | -0.0 | 0.0 | -1.0 | -0.8 | -0.7 | -0.8* | -0.9 | -0.9 | 0.4 | 0.3 | -0.7 | -0.7 | -0.3 | -0.4 |
| Inpatient | -1.6** | -1.6** | -1.8** | -2.0 | -1.2 | -1.2 | -2.1*** | -2.2*** | -2.4*** | 2.4* | -1.2 | 2.1 | 0.7 | 1.0 | 1.0 |
| Unplanned Returns | 0.6 | 0.5 | 0.2 | -8.0* | -7.2* | -8.0* | -1.2 | -1.5 | -1.8 | 1.6 | 1.3 | -0.1 | -2.7 | -2.4 | -1.5 |
|  | **Urban** | | | | | | | | | | | | | | |
| **Outcome** | 50% | 75% | 100% | 50% | 75% | 100% | 50% | 75% | 100% | 50% | 75% | 100% | 50% | 75% | 100% |
| ED Visits | 4.3 | 5.0 | 5.0 | -58.0*** | -56.9*** | -56.0*** | -28.0*** | -26.8*** | -25.2*** | 21.2* | 20.1* | 19.3 | -36.7*** | -33.4** | -39.9*** |
| Observation | 0.2 | 0.2 | 0.2 | -1.6*** | -1.6*** | -1.7*** | -0.9*** | -0.9*** | -0.9*** | 0.7 | 0.9 | 1.3* | -0.7 | -0.6 | -0.8 |
| Inpatient | -1.9*** | -1.8*** | -1.7*** | -4.8*** | -4.7*** | -4.7*** | -4.4*** | -4.4*** | -4.4*** | 0.6 | 0.6 | 0.1 | -3.1*** | -2.8*** | -2.8** |
| Unplanned Return | -0.6 | -0.6 | -0.7 | -8.4*** | -9.0*** | -9.5*** | -4.1*** | -4.3*** | -4.6*** | 2.2 | 2.7 | 3.0 | -4.9** | -5.0** | -7.2*** |

*p<0.01, **p<0.001, ***p<0.0001

Note: ED visit coefficients indicate magnitude per 100 person-years and all other coefficients indicate percentage point change
